# Succinate-Based Dietary Supplement for Menopausal Symptoms: A Pooled Analysis of Two Identical Randomized, Double-Blind, Placebo-Controlled Clinical Trials

**DOI:** 10.1155/2019/1572196

**Published:** 2019-10-31

**Authors:** Viktor E. Radzinsky, Yulia Uspenskaya, Lee P. Shulman, Irina V. Kuznetsova

**Affiliations:** ^1^Department of Obstetrics and Gynecology with a Course of Perinatology, The Peoples' Friendship University of Russia Medical Institute, Mikluho-Maklaya St. 8, 117198 Moscow, Russia; ^2^Department of Obstetrics and Gynecology, The I.M. Sechenov First Moscow State Medical University, Elanskogo Str. 2, bld. 1, Moscow, Russia; ^3^Department of Obstetrics and Gynecology, Feinberg School of Medicine of Northwestern University, 250 East Superior Street, Room 05-2174, Chicago, USA

## Abstract

**Background:**

To evaluate the efficacy of a succinate-based dietary supplement (SBDS; Amberen) in symptomatic menopausal women using a larger sample size derived by pooling data from two identical trials.

**Methods:**

Raw *data were pooled from two identical* randomized, multicenter, double-blinded, placebo-controlled, 90-day clinical trials. Women aged 42–60 years with mild to moderate vasomotor and psychosomatic menopausal symptoms were included (114 in the treatment group and 113 in the placebo group). Symptoms were assessed by the Greene Climacteric Scale and State-Trait Anxiety Inventory. Changes in body mass index, body weight, waist and hip circumferences, and plasma levels of follicle stimulating hormone, luteinizing hormone, estradiol, leptin, and apolipoproteins A1 and B were also evaluated.

**Results:**

SBDS use resulted in significant improvements in several endpoints including alleviation of 16 of 21 menopausal symptoms (*p* ≤ 0.05, Greene Scale) and a decrease in anxiety (*p* < 0.0001, State-Trait Anxiety Inventory) when compared to placebo. Significant reductions were observed in weight, body mass index, and waist and hip circumferences in the supplement cohort. Evaluation of physiological parameters showed a significant increase in serum estradiol levels compared to baseline (*p* < 0.0001) among users of the SBDS. Levels of follicle stimulating hormone and luteinizing hormone decreased slightly in both groups, without significant differences between the groups. Leptin levels decreased with statistical significance in the SBDS cohort compared to placebo (*p*=0.027). For those with initial leptin concentrations above the reference range, leptin decreased significantly in the SBDS group compared to the baseline (*p* < 0.0001) and to placebo (*p*=0.027).

**Conclusions:**

The pooled analysis reaffirms the outcomes from the individual trials. A nonhormonal, succinate-based dietary supplement is shown to relieve menopausal symptoms when compared to a placebo regimen in a randomized, double-blinded clinical trial.

## 1. Introduction

The physiological changes associated with the onset of menopause lead to a decrease in the quality of life for about 80% of women [1–3]. Besides the decrease in ovarian sex steroid production, the emergence of vasomotor, psychosomatic, and cognitive menopausal symptoms is also attributed to the age-related changes in the central nervous system (CNS) resulting from a reduced sensitivity to peripheral input signals and neurohormones' metabolism dysfunction [4–6].

Hormone therapy (HT) has been shown to be the most effective approach for the reduction of menopause-related vasomotor symptoms [7–9]. However, HT is but one pharmacotherapeutic strategy used to reduce vasomotor symptoms, along with lifestyle modifications, nutritional alterations, and increase in physical activity as well as other pharmaceutical and pharmacological interventions [8]. In situations when a woman has contraindications to the use of HT or declines to use it, her clinician should provide advice on alternative nonhormonal therapies for relief of menopausal symptoms.

Amberen, a proprietary combination of salts of succinic and fumaric acids, vitamin E, zinc, calcium, magnesium, L-glycine, and monosodium L-glutamate, is a nonprescriptive and nonhormonal dietary supplement that has been shown to reduce menopausal symptoms. Amberen was well-tolerated in a 4-week murine study, where older animals showed resumption of estral cycle and increased weight and calcium content of bone tissue. The effectiveness of this succinate-based dietary supplement (SBDS) to reduce menopause-related symptoms has been previously demonstrated in randomized, double-blind clinical trials [10–12]. While sufficient to demonstrate the product's efficacy, the previous trials were relatively small. This prompted the pooling of the data to analyze larger sample size, in order to better assess the supplement's efficacy in symptomatic menopausal women.

## 2. Materials and Methods

### 2.1. Design

Details of the two trials have been previously published [11, 12].

Two identical 90-day, randomized, double-blind, placebo-controlled trials of symptomatic menopausal women were conducted in Russia. The subjects were recruited by advertising at the research centers and doctors' offices. Trial 1 was conducted from August 4, 2014 (first patient screened), to April 22, 2015 (last patient completed), and randomized 104 subjects to 3 sites. Trial 2 was conducted from August 5, 2015 (first patient screened), to January 29, 2016 (last patient completed), and randomized 125 subjects to 3 sites.

The two trials were identical in design, protocol, randomization procedure, dosage, and trial procedures. In both trials, blood collection and clinical laboratory assays were done according to the same protocols, in the same laboratories, and on the same equipment. Both trials used the same version of the Greene Climacteric Scale and STAI questionnaires; the tests were administered in identical manner. All weight, height, and waist and hip circumference measurements were done with the same measuring techniques.

The 2014/2015 trial took place over summer, fall, winter, and spring, while the 2015/2016 trial was conducted over the course of summer, fall, and winter. The climacteric syndrome is not a seasonal pathology; thus, no correction coefficient was introduced in the pooled analysis. Since the two trials were identical in every aspect, the raw data could be pooled for analysis; meta-analysis coefficient is equal to one for all parameters.

The trials enrolled symptomatic postmenopausal (12 months amenorrhea, STRAW + 10 classification [13]) women between 42 and 60 years with mild to moderate vasomotor and psychosomatic menopausal symptoms (Table 1). The total number of subjects in the pooled analysis was 114 in the treatment group (SBDS) and 113 in the control group (placebo). Two subjects in the placebo group from the first trial withdrew from the study due to medical causes unrelated to the intervention. None of the subjects withdrew from the second trial.

Subjects were excluded if any of the following were present at screening: oncological conditions, endocrinopathic conditions, psychiatric diseases, chronic somatic illnesses requiring therapies that could impact the trials' outcomes (e.g., use of antidepressants for smoking cessation), use of hormonal therapies less than 6 months prior to the screening, or the use of dietary supplements or medications that could affect climacteric symptoms less than 1 month prior to enrolling in the trials.

The following information was collected from all subjects at study initiation: demographic data, general health information, and general and gynecological medical history. Anthropometric measurements (height, body weight, and waist and hip circumferences) and vital signs were recorded; subjects underwent general and gynecological exams. Standard laboratory evaluation included routine hematological and biochemical assays, urinalysis (Appendix A), mammogram, and ultrasound of lower pelvic organs. The complete schedule of procedures is shown in Table 2.

### 2.2. Ethics Approval and Consent to Participate

All procedures performed in studies involving human participants were in accordance with the ethical standards of the institutional and/or national research committee and with the 1964 Helsinki declaration and its later amendments or comparable ethical standards. Informed consent was obtained from all individual participants included in the study. The protocols of both trials had been approved by a local IRB.

### 2.3. Primary Outcome Measures

To evaluate the SBDS's overall efficacy with regard to vasomotor and psychosomatic climacteric symptoms, the Greene Climacteric Scale [14, 15] and State-Trait Anxiety Inventory (STAI) [16] were employed at baseline, 30th, 60th, and 90th days of the trials. The Greene Scale is comprised of 21 symptoms, each to be ranked as absent or as one of three levels of severity. The mean scores of each symptom at baseline were compared to the placebo at the end of the trial. Total Greene scores were not evaluated as the individual symptoms are more informative in evaluating efficacy of the supplement. STAI evaluates personal or trait anxiety (TA), situational or state anxiety (SA), and an integral parameter of the actual anxiety (AA) [17–19].

### 2.4. Secondary Outcome Measures

Weight, body mass index (BMI), waist and hip circumferences, and levels of FSH, LH, estradiol, leptin, and apolipoproteins A1 and B were evaluated as secondary endpoints. Weight measurements, waist and hip circumferences, and serum levels of FSH, LH, and estradiol were recorded at baseline and at the 30th, 60th, and 90th days of the trials. Levels for leptin and apolipoproteins A1 and B were evaluated at baseline and on the 90th day of the trials. Fasting blood samples were collected in the morning and analyzed by manufacturer's protocols as follows: estradiol with high sensitivity radioimmune assay (RIA) (Immunotech A.S., Czech Republic, sensitivity 6 pg/ml); FSH with high sensitivity RIA (Immunotech A.S., Czech Republic, sensitivity 0.17 IU/L); LH with high sensitivity RIA (Immunotech A.S., Czech Republic, sensitivity 0.16 IU/L); leptin with high sensitivity RIA (RIA-1624, DRG instruments GmbH, Germany); and apolipoproteins A1 and B with immunoturbidimetric assays (cobas® 6000, Roche).

### 2.5. Randomization and Intervention

Randomization was accomplished using an automated method of random number generation. Subjects in the treatment group took the SBDS (the product's ingredients are separated into two capsules for stability: one white capsule with ammonium succinate, 200 mg, and one orange capsule (200 mg) with calcium disuccinate, monosodium-L-glutamate, glycine, magnesium disuccinate, zinc difumarate, and tocopherol acetate) once a day with a meal, typically at or after breakfast, for 90 days. Subjects in the placebo group received capsules with high purity corn starch; these capsules were identical in appearance, taste, and smell to the supplement capsules. A physician evaluated all subjects on a monthly basis for changes in the aforementioned parameters beginning at the initiation to completion of the trial.

### 2.6. Safety Monitoring

Subjects underwent monthly evaluations (with vital signs measurements) at the research centers and were evaluated for any adverse events. Hematological assays, biochemical assays, and urinalysis were performed at baseline and at the conclusion of the study (Appendix A).

### 2.7. Statistical Analysis

Pooled data from the two studies were analyzed using MS Office 2010 and STATISTICA 12. Average (***M***), standard error (*m*), and deviation (*σ*), dispersion interval (minimum and maximum) were all calculated for quantitative parameters. Frequency of qualitative parameters was expressed in percent (%). All measured parameters were evaluated for normal distribution using Shapiro–Wilk, Lilliefors, and Kolmogorov–Smirnov tests. In order to describe quantitative parameters with nonparametric characteristics, the following were used: volume sample, median and average, maximum, minimum, and 95% confidence interval. Comparative analysis of efficacy parameters (quantitative variables) was performed by comparing averages in the treatment and control groups. Comparison of averages for each parameter in the two groups was done using Mann–Whitney *U* test (nonparametric statistics) or Student's *t*-test (for normal distribution). The Friedman test was used for analysis within each group. For calculations of the Greene Climacteric Scale scores between the groups, *χ*^2^ test was used. Differences were considered statistically significant at a *p* < 0.05 (95% confidence level).

Contribution of outliers to the median values was assessed using standard analysis.

For some parameters, statistically significant differences were found at baseline between the SBDS and placebo groups (Greene questions 3, 9, 15, 19, and 21; trait and actual anxiety; estradiol). These differences disappeared by the 30th day of the trial and thus this time point was taken as a baseline in the analysis of those parameters.

## 3. Results

### 3.1. Baseline Assessment

A total of 227 women completed the two randomized placebo-controlled clinical trials. All study subjects were Caucasians of Russian ancestry. Mean age was not significantly different between the SBDS (52.0 ± 4.68 years) and placebo (51.6 ± 4.64 years) cohorts. Approximately 90% of the study subjects were within 3 years of menopause onset and 10% were menopausal for longer than 3 years, with no more than 5.5 from the onset. The two cohorts were comparable with no significant differences in any of the demographic characteristics. Anthropometric parameters, results of the blood analyses and urinalyses, levels of apolipoproteins A1 and B, and leptin also did not differ significantly between the two groups at study initiation.

Results of the initial Greene Climacteric Scale assessments were mostly similar between the two cohorts. The categories that were not similar at baseline included “difficulty sleeping”, “sadness or depression”, “hot flashes”, and “lack of sex drive,” symptoms that occurred more frequently in the SBDS cohort (Table 3). STAI trait and actual anxiety scores indicated more severe anxiety levels in the SBDS cohort at baseline (Table 4).

### 3.2. Greene Climacteric Scale Evaluation of Menopausal Symptoms

Analysis of the Greene Climacteric Scale data showed a significant decrease in the number of complaints and degree of bother for most symptoms in the SBDS cohort (Table 3), except for “numbness or tingling in some body parts”, “numbness of hands and feet”, and “difficulty breathing” in which no significant changes were observed. In the placebo group, there was a significant increase observed in the number and degree of bother for “difficulty sleeping,” “feeling tired and lacking energy,” “loss of interest in most things,” “sadness or depression,” “crying spells,” “irritability,” “feeling pressure or tightness in the head or other body parts,” “headaches,” “muscle and joint pain,” “hot flashes,” “night sweats,” and “lack of sex drive” at the end of the study. Comparative analysis between the two cohorts showed statistically significant differences favoring the SBDS cohort in the following symptoms at study conclusion: “heart palpitation,” “difficulty sleeping,” “sadness or depression,” and “hot flashes.” By the end of the study, there was also a significant improvement in the overall clinical presentation (decrease in the number of complaints and symptoms' severity) in the SBDS group compared to placebo in the following symptoms: “heart palpitation,” “feeling tense or nervous,” “difficulty sleeping,” “increased excitability,” “difficulty concentrating,” “feeling tired or lacking,” “loss of interest in most things,” “sadness or depression,” “crying spells,” “irritability,” “headaches,” “muscle and joint pain,” “hot flashes,” “night sweats,” and “lack of sex drive.”

### 3.3. STAI Evaluation of Anxiety

After one month of treatment, all anxiety parameters were found to have significant decrease in the SBDS group, while showing a significant increase in the placebo group (supplement: SA from −1.0 to −5.0; TA from 14.0 to 10.0; AA from 48.0 to 40.0; placebo: SA from −3.0 to −2.0; TA from 11.0 to 13.0; AA from 42.0 to 44.0). Starting from the second month of the treatment and until the end of the trial, all anxiety parameters were significantly different and showed a more favorable outcome for the SBDS cohort when comparing the two groups (supplement: SA from −5.0 to −8.0; TA from 10.0 to 4.0; AA from 40.0 to 30.0; placebo: SA from −2.0 to −1.0; TA from 13.0 to 16.0; AA from 44.0 to 49.0).

### 3.4. Anthropometric Parameters Evaluation

No significant differences between the study cohorts were observed in the anthropometric parameter analysis with the exception of weight. The mean baseline weight did not differ significantly between the SBDS (76.65 ± 11.86 kg) and placebo (76.46 ± 14.96 kg) groups (*p*=0.918). However, by the end of the study, the weight of subjects in the SBDS group decreased by 3.5 kg (4.57%) and was 73.6 ± 10.71 kg, whereas, in the placebo group, body weight increased by 0.82 kg (1.07%) and was 77.28 ± 14.95 kg, a significant difference (*p*=0.033). The rest of the anthropometric parameters showed a similar reduction within the SBDS group and a small increase in the placebo group. BMI was 28.56 kg/m^2^ ± 4.81 at study initiation and after treatment was 27.41 kg/m^2^ ± 4.18 kg/m^2^ (4.03% decrease) in the SBDS group. In the placebo group, baseline BMI was 28.34 kg/m^2^ ± 5.83 and after treatment was 28.64 kg/m^2^ ± 5.81 (1.06% increase). With regard to waist circumference, baseline was 88.49 cm ± 11.85 and after study completion was 85.14 cm ± 10.24 (3.79% decrease) in the SBDS group baseline compared to 86.7 cm ± 13.78 at study initiation and after treatment was 87.67 cm ± 13.76 cm in the placebo group (1.06% increase). By the end of the study, hip circumference decreased by 3.1 cm in the SBDS group (the measurements were 104.99 cm ± 11.32 at baseline and 101.89 cm ± 9.86 after the trial). In the placebo group, hip circumference remained essentially unchanged: 104.13 cm ± 11.2 at baseline and 104.35 ± 11.71 by the end of the study.

### 3.5. Plasma Hormone Levels and ApoA1/B Evaluation

Evaluation of plasma hormone levels at baseline showed the serum estradiol level (Appendix B) to be significantly higher in the placebo group (Table 5, 42.3 pg/ml compared to 35.7 pg/ml in the supplement group). However, after just one month of treatment, subjects in the SBDS group showed a significant increase in estradiol concentration, with the serum level being significantly higher than that observed in the placebo group (48.9 pg/ml in the supplement and 41.1 pg/ml in the placebo group). In the treatment group at the next visit evaluation point, an increased level of estradiol continued to be observed. Plasma estradiol levels in women in the SBDS cohort were significantly higher at the end of the study compared to baseline levels and the placebo group, with the latter showing a decrease in the estradiol levels (at the 90th day in the supplement group = 58.0 pg/ml, placebo = 39.9 pg/ml).

Levels of FSH and LH were observed to decrease in both groups, with a statistically significant reduction observed in the SBDS group. However, the comparative analysis of the two study groups showed no significant differences in FSH and LH levels (Table 5). Concentrations of leptin in the SBDS group were characterized by a nonsignificant trend toward decreasing levels; however, by the end of the trial, there was a significant decrease in leptin levels in the SBDS group compared to the placebo group.

There were no significant differences between the study groups in the average apolipoprotein levels A1 and B or their ratios or their comparative analyses (SBDS group, ApoA1 baseline = 1.65 g/L, 90 days = 1.64 g/L; ApoB baseline = 0.97 g/L, 90 days = 0.97 g/L; placebo group, ApoA1 baseline = 1.62 g/L, 90 days = 1.57 g/L; ApoB baseline = 0.93 g/L, 90 days = 0.96 g/L).

### 3.6. Leptin, Leptin Resistance, and BMI Evaluation

In consideration of the anthropomorphic changes observed in this study, we conducted an analysis of the changes in the leptin levels in those subjects for whom baseline levels of leptin were above the reference range (above 11.09 ng/ml) (91 subjects in the SBDS group and 84 subjects in the placebo group) in order to study the effects of SBDS on women who may have metabolic dysfunctions. In the supplement group, leptin levels decreased from 16.5 ng/ml to 14.1 ng/ml (*p* < 0.0274) with no significant changes being observed in the placebo group (baseline 15.4.0 ng/ml and 15.3 ng/ml by the end of the study). There was no difference in the baseline values of the two study cohorts (*p*=1.0), but by the end of the study, differences in leptin levels comparing the two groups showed a significant decrease in the SBDS cohort compared to placebo (*p*=0.0006). Ratio of leptin to BMI, which can reflect leptin resistance, decreased from 0.68 to 0.54 (*p* < 0.0001) in the SBDS cohort by the end of the study. This drop was statistically significant (*p*=0.029) compared to the placebo group, where leptin to BMI ratio was 0.65 at study commencement and 0.64 at study conclusion.

### 3.7. Safety Outcomes

With regard to safety outcomes, we found no serious adverse events to have occurred in either cohort. Patients reported no side effects during the performance of both studies. Hematological parameters, urinalysis, and vital signs were monitored during the trial and showed no differences between SBDS and placebo groups.

## 4. Discussion

Our study, utilizing pooled data from 227 subjects in the two randomized, placebo-controlled clinical trials, demonstrates the salutary impact (both statistically significant and clinically relevant) of the recommended use of the SBDS regimen on menopausal symptoms. One of the goals of the individual trials and this pooled analysis was to evaluate the SBDS regimen effect, if any, on the quality of life of symptomatic menopausal women. To this end, the Greene Climacteric Scale was chosen to be used to evaluate study subjects as it was felt to provide a more comprehensive overview of the overall impact of the SBDS regimen. The Greene Climacteric Scale is a validated tool commonly used in clinical trials evaluating impact of an intervention on menopausal symptoms [20–23]. Recently, a number of publications have highlighted that the simple measure of frequency and severity of vasomotor symptoms may not provide an overarching assessment of the overall efficacy of an intervention since the frequency/severity of vasomotor symptoms do not necessarily correlate with the degree of bother and quality of life of symptomatic menopausal women [24, 25].

Given our two evaluation parameters—significant reduction of symptoms' bother within the SBDS group and significant difference in the symptoms' bother between the SBDS and placebo groups at the end of the study—we can conclude that the use of the supplement demonstrates significant beneficial effects on the following Greene Climacteric Scale symptoms: “heart palpitations,” “feeling tense or nervous,” “difficulty sleeping,” “increased excitability,” “difficulty concentrating,” “feeling tired or lacking energy,” “loss of interest in most things,” “sadness or depression,” “crying spells,” “irritability,” “headaches,” “muscle and joint pain,” “hot flashes,” “night sweats,” and “lack of sex drive.” The positive effect of the SBDS regimen on psychiatric functions is likewise demonstrated by a significant decrease in anxiety among the SBDS group with a corresponding increase in anxiety markers among women in the placebo group.

Presently, the mechanism of action of the SBDS regimen has not been entirely elucidated. Some may point to the observed increase in serum estradiol levels among SBDS users as the sole or major reason for the clinical benefits observed. However, it is important to recognize that the magnitude of increase in serum estradiol levels in this study is considerably less than that observed in conventional hormone therapy trials [26] and that certain benefits, such as anxiety reduction, have not been historically associated with postmenopausal estrogen use. While the etiology of the observed increase in serum estradiol levels among SBDS users is not currently known, it is likely an indirect effect as the components of the SBDS regimen are not known to be involved in the hormone synthesis pathway. It is possible that SBDS acts by supporting communication along the hypothalamic-pituitary-ovarian axis. Succinate anions act as a metabolic intermediate and a signaling molecule [27]. Succinate provides energy supply and catecholamine-like influence on the hypothalamus, which increases the organ's sensitivity to input signals from peripheral endocrine glands [28].

The increase in serum estradiol levels observed among SBDS users was not associated with the ingestion of any estrogenic compound. As such, we are unable to associate these results with any adverse clinical outcomes associated with postmenopausal use of therapies containing exogenous estrogen. Nonetheless, women who should not use estrogen (e.g., with history of estrogen-positive breast cancer) may not wish to use the SBDS regimen for the relief of menopausal symptoms.

One possible mechanism of action for the clinical benefits attributed to SBDS is related to estrogen, but not solely as a process that increases serum levels. Estrogen is the most important regulator of energy metabolism in the CNS, where it targets neuronal mitochondria. Deficit in estrogen stimulation leads to a decrease in energy production in the neurons; this in turn causes a number of events—from inability to regenerate, receive, and conduct nerve impulses to neuronal cell [29, 30]. Vasomotor instability, psychosomatic symptoms, and sleep disturbances, which can result from the above-mentioned neuronal dysregulation, potentiate one another and add to the development of anxiety and depressive symptoms cognitive dysfunctions and an increased risk of metabolic syndrome [31, 32]. Perhaps the use of ammonium succinate, which also targets mitochondria, is able to restore some of the energy deficit that resulted from the low estrogen levels in the CNS and thus leads to an improvement in a variety of menopausal symptoms [33].

Another finding in our study that has not been associated with conventional hormone therapy is weight loss. Our study yielded clinical data on the positive effect of the SBDS use on overall metabolism, specifically, a small but significant weight reduction in subjects in the SBDS cohort compared to the placebo group, along with a significant drop in leptin levels in subjects with elevated levels of the hormone at baseline. We also observed a decrease in the BMI/leptin ratio, a finding that we believe may reflect an improvement in the tissue sensitivity to leptin, potentially resulting in a positive impact on overall metabolism.

### 4.1. Study Strengths and Limitations

As with all studies, ours is characterized by strengths and weaknesses. Our greatest strength is the high retention rate and high adherence (only 2 patients withdrew from the first study) to the protocol of the studies. However, this strength may actually highlight a weakness of our study; namely, the homogeneity of the study population. It is possible that these findings may not be similarly observed in studies of more diverse communities or among women who are not as adherent to the recommended use of SBDS as were most of the women in the two trials. In addition, the relatively short intervention study period of these trials provides no information on the benefits of long-term use of the SBDS regimen or on the safety profile associated with long-term use. Further study is clearly needed to better assess the benefits and risks of long-term use of the SBDS regimen.

## 5. Conclusion

Our pooled study affirms that this SBDS can provide a beneficial impact on a wide variety of adverse physical and psychological symptoms experienced by many women transitioning through the menopause.

The subjects took SBDS or placebo for 90 days and were evaluated by a physician monthly. A follow-up phone call to assess any adverse events took place at 120 days (30 days after the last dose of the SBSD/placebo).

## Figures and Tables

**Table 1:** Demographic characteristics at baseline.

Parameter	SBDS cohort (*n* = 114)	Placebo cohort (*n* = 113)
Mean age, years	51.97	51.6
Mean weight, kg	76.76	76.43
Natural menopause (12 months amenorrhea), number of patients	114	112
Surgical menopause, number of patients	0	1

**Table 2:** Schedule of tests and procedures.

Data collected	Trial days			
	Baseline	30 days	60 days	90 days
Greene Climacteric Scale	✓	✓	✓	✓
State-trait anxiety inventory	✓	✓	✓	✓
Waist and hip circumference (cm), weight (kg)	✓	✓	✓	✓
Estradiol, FSH, LH	✓	✓	✓	✓
Vital signs	✓	✓	✓	✓
Gynecological exam	✓			✓
Hematological assay	✓			✓
Biochemical assay	✓			✓
Urinalysis	✓			✓
Leptin, apolipoproteins А1 and В	✓			✓

**Table 3:** Greene Climacteric Scale results.

	Group	*n*	Number (%) of women with symptoms		
			Before treatment	After treatment	*p*
1. Heart palpitation	Supplement	114	80 (70.1%)	55 (48.2%)	**0.0008**
	Placebo	113	76 (67.3%)	76 (67.3%)	0.7194
	*p*		0.2980	**0.0215**	
2. Feeling tense or nervous	Supplement	114	92 (80.7%)	86 (75.4%)	**0.0003**
	Placebo	113	90 (79.6%)	91 (80.5%)	0.9655
	*p*		0.9811	**0.0012**	
3. Difficulty sleeping	Supplement	114	98 (85.9%)	52 (45.6%)	**≤0.001**
	Placebo	113	82 (72.6%)	91 (80.5%)	**0.0067**
	*p*		**0.0007**	**≤0.001**	
4. Increased excitability	Supplement	114	86 (75.4%)	55 (48.2%)	**≤0.001**
	Placebo	113	82 (72.6%)	83 (73.5%)	0.8861
	*p*		0.6274	**≤0.001**	
5. Panic attacks	Supplement	114	41 (36.0%)	27 (23.7%)	**0.0433**
	Placebo	113	35 (31.0%)	30 (26.5%)	0.6298
	*p*		0.8248	0.2745	
6. Difficulty concentrating	Supplement	114	82 (71.9%)	35 (30.7%)	**≤0.001**
	Placebo	113	72 (63.7%)	85 (75.2%)	0.2535
	*p*		0.4192	**≤0.001**	
7. Feeling tired or lacking energy	Supplement	114	106 (93.0%)	54 (47.4%)	**≤0.001**
	Placebo	113	101 (89.4%)	105 (93.0%)	0.0490
	*p*		0.0767	**≤0.001**	
8. Loss of interest in most things	Supplement	114	69 (60.5%)	35 (30.7%)	**≤0.001**
	Placebo	113	52 (46.0%)	66 (58.4%)	0.2947
	*p*		0.1277	**≤0.001**	
9. Sadness or depression	Supplement	114	99 (86.8%)	40 (35.1%)	**≤0.001**
	Placebo	113	77 (68.1%)	88 (78.1%)	**0.0087**
	*p*		**0.0043**	**≤0.001**	
10. Crying spells	Supplement	114	63 (55.3%)	44 (38.6%)	**0.0012**
	Placebo	113	62 (55.0%)	63 (55.8%)	0.7053
	*p*		0.9988	**0.0080**	
11. Irritability	Supplement	114	104 (91.2%)	50 (44.0%)	**≤0.001**
	Placebo	113	92 (81.4%)	98 (86.7%)	0.0709
	*p*		0.1970	**≤0.001**	
12. Dizziness or fainting	Supplement	114	54 (47.4%)	40 (35.1%)	**0.0196**
	Placebo	113	40 (35.4%)	35 (31.0%)	0.7467
	*p*		0.3405	0.2626	
13. Feeling pressure or tightness in the head or other body parts	Supplement	114	73 (64.0%)	46 (40.4%)	**0.0010**
	Placebo	113	60 (53.1%)	57 (50.4%)	0.9535
	*p*		0.4026	0.1534	
14. Numbness or tingling in some body parts	Supplement	114	65 (57.0%)	54 (47.9%)	0.0748
	Placebo	113	68 (60.2%)	65 (57.5%)	0.8573
	*p*		0.4835	**0.0327**	
15. Headaches	Supplement	114	99 (86.8%)	62 (54.4%)	**≤0.001**
	Placebo	113	93 (82.3%)	93 (82.3%)	0.6250
	*p*		0.04996	**≤0.001**	
16. Muscle and joint pain	Supplement	114	97 (85.1%)	60 (52.6%)	**≤0.001**
	Placebo	113	100 (88.5%)	91 (80.5%)	0.3175
	*p*		0.0574	**≤0.001**	
17. Numbness of hands and feet	Supplement	114	49 (43.0%)	41 (36.0%)	0.4467
	Placebo	113	42 (37.2%)	40 (35.4%)	0.8413
	*p*		0.5336	0.7042	
18. Difficulty breathing	Supplement	114	45 (39.5%)	35 (30.7%)	0.2542
	Placebo	113	36 (32.0%)	37 (32.7%)	0.6667
	*p*		0.6805	0.9836	
19. Hot flashes	Supplement	114	101 (88.6%)	50 (44.0%)	**≤0.001**
	Placebo	113	86 (76.1%)	91 (80.5%)	**0.0137**
	*p*		**0.0158**	**≤0.001**	
20. Night sweats	Supplement	114	92 (80.7%)	46 (40.4%)	**≤0.001**
	Placebo	113	85 (75.2%)	87 (77.0%)	0.2737
	*p*		0.4847	**≤0.001**	

21. Lack of sex drive	Supplement	114	92 (80.7%)	39 (34.2%)	**≤0.001**
	Placebo	113	76 (67.3%)	88 (78.0%)	0.1692
	*p*		**0.0089**	**≤0.001**	

Number of symptomatic subjects before and after treatment (those that marked that each symptom bothered them at least “a little” as per Greene Climacteric Scale). Bold numbers indicate statistically significant *p* values.

**Table 4:** State-trait anxiety inventory results.

Anxiety	Time	Supplement *n* = 114	Placebo *n* = 113	*p* ^a^
SA (state anxiety)	Initial	−1.0 [−7.0, 7.0]	−3.0 [−10.0, 3.0]	0.0583^*∗*^
	30 days	−5.0 [−9.0, 1.0]	−2.0 [−7.0, −3.0]	**0.0495**
	60 days	−7.0 [−11.0, −3.0]	0.0 [−6.0, 4.0]	**≤0.001**
	90 days	−8.0 [−13.0, −4.0]	−1.0 [−7.0, −4.0]	**≤0.001**
	*p* ^b^	**<0.05**	**<0.05**	

TA (trait anxiety)	Initial	14.0 [8.0, 22.0]	11.0 [7.0, 17.0]	**0.0125** ^*∗*^
	30 days	10.0 [5.0, 15.0]	13.0 [8.0, 17.0]	**0.0212**
	60 days	7.0 [4.0, 10.0]	14.0 [9.0, 21.0]	**≤0.001**
	90 days	4.0 [1.0, 8.0]	16.0 [9.0, 20.0]	**≤0.001**
	*p* ^b^	**<0.05**	**<0.05**	

АA (actual anxiety)	Initial	48.0 [37.0, 61.0]	42.0 [33.0, 53.0]	**0.0146** ^*∗*^
	30 days	40.0 [32.0, 50.0]	44.0 [37.0, 55.0]	**0.0110**
	60 days	34.0 [28.0, 41.0]	48.0 [39.0, 57.0]	**≤0.001**
	90 days	30.0 [24.0, 38.0]	49.0 [36.0, 57.0]	**≤0.001**
	*p* ^b^	**<0.05**	**<0.05**	

Indicated values are (Me [*Q*1, *Q*3]). *p*^a^: supplement versus placebo (Mann–Whitney test, *t-*test^*∗*^); *p*^b^: initial versus 90 days (Friedman test). Bold numbers indicate statistically significant *p* values.

**Table 5:** Plasma hormone concentrations.

Hormones	Time	Supplement *n* = 114	Placebo *n* = 113	*p* ^a^
Estradiol (pg/ml)	Initial	35.7 [28.4, 47.1]	42.3 [29.1, 52.6]	**0.0187**
	30 days	48.9 [35.3, 59.9]	41.1 [30.1, 52.8]	**0.0123**
	60 days	54.0 [42.0, 65.0]	40.4 [28.1, 50.4]	**≤0.001**
	90 days	58.0 [46.1, 58.6]	39.9 [27.2, 50.2]	**≤0.001**
	*p* ^b^	**<0.05** ^*∗*^	**<0.05** *∗*	

FSH (mIU/ml)	Initial	57.2 [40.6, 74.1]	51.5 [37.3, 68.4]	0.0938
	30 days	53.9 [40.9, 70.7]	52.0 [39.7, 70.1]	0.9291
	60 days	51.2 [38.1, 65.2]	50.8 [38.8, 71.3]	0.4594
	90 days	48.2 [37.1, 62.8]	50.2 [38.7, 68.5]	0.3131
	*p* ^b^	**<0.05** ^*∗*^	>0.05^*∗*^	

LH (mIU/ml)	Initial	31.1 [22.9, 38.9]	29.2 [21.8, 35.7]	0.1740
	30 days	30.5 [24.0, 39.0]	29.9 [22.2, 36.1]	0.4118
	60 days	29.9 [21.4, 35.4]	28.9 [22.7, 35.2]	0.8532
	90 days	28.3 [20.8, 34.1]	28.4 [22.0, 35.2]	0.6492
	*p* ^b^	**<0.05** ^*∗*^	>0.05^*∗*^	

Leptin (ng/ml) (0.5–13.8)	Initial	16.5 [11.9, 23.8]	15.4 [10.5, 24.2]	0.4509
	90 days	14.1 [10.5, 18.2]	15.3 [11.3, 27.8]	**0.0274**
	*p* ^b^	>0.05^*∗*^	>0.05^*∗*^	

Indicated values are (Ме [*Q*_1_, *Q*_3_]). *p*^a^: supplement versus placebo (Mann–Whitney test); *p*^b^: initial versus 90 days (^*∗*^Friedman test, Mann–Whitney test). Bold numbers indicate statistically significant *p* values.

## Data Availability

The datasets used and/or analyzed during the current study are available from the corresponding author on request.

## Appendix

### A. Hematological and Biochemical Assays and Urinalysis Parameters

Hematological assay: hemoglobin, erythrocytes, color indicator, platelets, leukocytes, and leukocyte composition (segmented neutrophils, banded neutrophils, eosinophils, monocytes, and basophils).

Biochemical assay: total protein, total bilirubin, aspartate aminotransferase, alanine aminotransferase, creatinine, glucose, and cholesterol.

Urinalysis: color, opacity, specific weight, pH, glucose, ketone bodies, leukocytes, erythrocytes, and microscopic analysis of the sedimentation.

### B. Standard Laboratory Estradiol Ranges

  Follicular: 12.5–166 pg/ml
  Ovulatory: 85.8–498.0 pg/ml
  Luteal: 43.8–211 pg/ml
  Postmenopausal: 5–54.7 pg/ml (with the first year following FMP: 20–80 pg/ml)

## Abbreviations

AA: Actual anxiety
BMI: Body mass index
CNS: Central nervous system
FSH: Follicle stimulating hormone
HT: Hormone therapy
LH: Luteinizing hormone
SA: State anxiety
SBDS: Succinate-based dietary supplement
STAI: State-trait anxiety inventory
TA: Trait anxiety.
